# Functional Polymorphisms of the *ABCG2* Gene Are Associated with Gout Disease in the Chinese Han Male Population

**DOI:** 10.3390/ijms15059149

**Published:** 2014-05-22

**Authors:** Danqiu Zhou, Yunqing Liu, Xinju Zhang, Xiaoye Gu, Hua Wang, Xinhua Luo, Jin Zhang, Hejian Zou, Ming Guan

**Affiliations:** 1 Department of Laboratory Medicine, Jinshan Hospital, Fudan University, Shanghai 201508, China; E-Mail: zzdanqiu@163.com; 2 Department of Laboratory Medicine, Renhe Hospital, Baoshan District, Shanghai 200443, China; E-Mail: Liuyunqing@163.com; 3 Central Laboratory, Huashan Hospital, Fudan University, 12 Central Urumqi Road, Shanghai 200040, China; E-Mails: pp_dou@163.com (X.Z.); gxiaoye@126.com (X.G.); fdeduwh@126.com (H.W.); 4 Department of Clinical Laboratory, Taizhou Municipal Hospital, Taizhou 318000, Zhejiang, China; E-Mails: Luoxinhua@163.com (X.L.); zhangjin_jhb@163.com (J.Z.); 5 Department of Rheumatology, Huashan Hospital, Fudan University, Shanghai 200040, China; E-Mail: hejianzou@gmail.com

**Keywords:** gout, uric acid, polymorphism, ABCG2

## Abstract

**Background:**

Gout is a common type of arthritis that is characterized by hyperuricemia, tophi and joint inflammation. Genetic variations in the *ABCG2* gene have been reported to influence serum uric acid levels and to participate in the pathogenesis of gout, but no further data have been reported in the Han Chinese population.

**Methods:**

Peripheral blood DNA was isolated from 352 male patients with gout and 350 gout-free normal male controls. High-resolution melting analysis and Sanger sequencing were performed to identify the genetic polymorphisms V12M, Q141K and Q126X in the *ABCG2* gene. Genotype and haplotype analyses were utilized to determine the disease odds ratios (ORs). A prediction model for gout risk using ABCG2 protein function was established based on the genotype combination of Q126X and Q141K.

**Results:**

For Q141K, the A allele frequency was 49.6% in the gout patients and 30.9% in the controls (OR 2.20, 95% confidence interval (CI): 1.77–2.74, *p* = 8.99 × 10^−13^). Regarding Q126X, the T allele frequency was 4.7% in the gout patients and 1.7% in the controls (OR 2.91, 95% CI: 1.49–5.68, *p* = 1.57 × 10^−3^). The A allele frequency for V12M was lower (18.3%) in the gout patients than in the controls (29%) (OR 0.55, 95% CI 0.43–0.71, *p* = 2.55 × 10^−6^). In the order of V12M, Q126X and Q141K, the GCA and GTC haplotypes indicated increased disease risk (OR = 2.30 and 2.71, respectively). Patients with mild to severe ABCG2 dysfunction accounted for 78.4% of gout cases.

**Conclusion:**

The *ABCG2* 126X and 141K alleles are associated with an increased risk of gout, whereas 12M has a protective effect on gout susceptibility in the Han Chinese population. ABCG2 dysfunction can be used to evaluate gout risk.

## 1. Introduction

Gout is one of the most common forms of arthritis [1,2] and accounts for nearly 4 million outpatient visits every year in the US [3]. Gout also imposes a substantial physical and economic burden on patients [4]. The disease is characterized by joint pain, inflammation and painful tophi and can lead to joint destruction and disability if untreated [2]. Epidemiological studies from several countries have suggested that the prevalence and incidence of gout are increasing [2]. Gout and hyperuricemia are associated with other common diseases, including hypertension [2], coronary artery diseases [5] and kidney diseases [6]. Major advances in our understanding of the pathogenesis and treatment of gout have been made over the past decade. Key highlights include the identification of genetic and environmental risk factors for gout. Genetic studies have demonstrated that serum uric acid levels are highly heritable [7]. Several urate transporters that influence serum uric acid levels have been identified in recent genome-wide association studies, mostly in populations of European descent [8–10].

The ATP-binding cassette (ABC) transporter, subfamily G, member 2 gene *ABCG2*/*BCRP* is located in a gout-susceptibility locus on chromosome 4q, which was previously identified in a genome-wide linkage study of gout [11]. The role of *ABCG2* as a urate transporter with mutations leading to hyperuricemia and gout was recently confirmed [12]. Human genetic analyses and animal model studies have demonstrated that ABCG2 dysfunction plays an important role in the pathogenesis of hyperuricemia [13]. Sequencing the *ABCG2* gene from human samples has revealed over 80 different naturally occurring sequence variations [14], several of which have been shown to result in proteins with functional alterations. Among these alleles, *ABCG2* 141K is associated with low levels of *ABCG2* expression and reduces the ATP-dependent transport of urate compared with the wild-type gene [15]. In addition, 126X has been demonstrated to impair the expression of active *ABCG2* and nearly eliminate transport activity [16]. *ABCG2* 12M has also been reported to induce the apical plasma membrane delocalization of ABCG2 and to produce a protein with a significantly reduced ability to transport several drugs [17]. The association between these three common *ABCG2* single-nucleotide polymorphisms (SNPs) and gout has not been thoroughly characterized in the Han Chinese male population.

Furthermore, the SNPs Q141K and Q126X in the human *ABCG2* gene have recently been recognized as clinical biomarkers to assess hyperuricemia and gout. Thus, a rapid method for detecting these mutations would be highly desirable.

High-resolution melting (HRM) is used as a simple and reliable technology for genotyping. This method enables researchers to rapidly detect and categorize genetic mutations, such as SNPs, and to identify new genetic variants without sequencing. In the present study, we developed an HRM assay to detect three functional SNPs (Q141K, V12M and Q126X) and then assessed the genetic association of those SNPs in the *ABCG2* gene with gout to investigate the association between ABCG2 dysfunction and gout risk in a Han Chinese male population.

## 2. Results

### 2.1. Distribution of ABCG2 Genotypes

**Figure 1.**
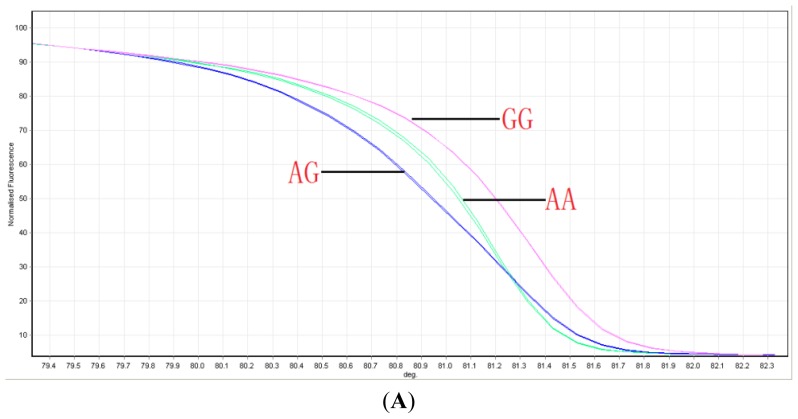

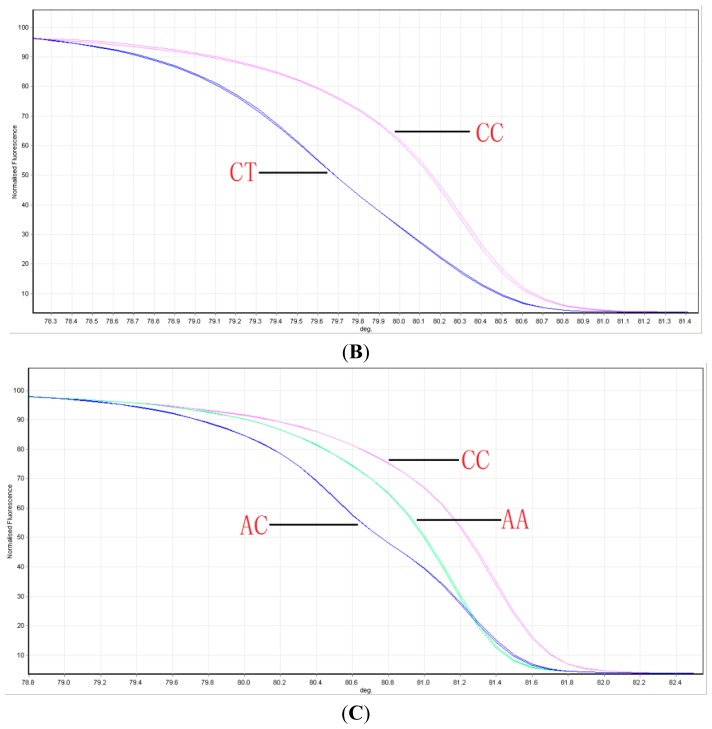
Melting curves of SNP genotypes in the *ABCG2* gene. The three groups are well distinguished: (**A**) V12M; (**B**) Q126X; and (**C**) Q141K.

The genotype assignments of the three SNPs were determined via HRM curves using the sequenced samples as control genotypes. The studied SNPs were successfully genotyped using HRM analysis, as shown in Figure 1. The results obtained from the DNA sequencing analysis confirmed the reliability of the HRM assay.

The genotype and allelic frequencies of the three SNPs (Q141K, V12M and Q126X) among the cases and controls were in Hardy-Weinberg equilibrium for all of the polymorphisms analyzed. For Q141K, the A allele was found on 49.6% of the chromosomes from the gout patients compared with 30.9% of the chromosomes from the controls (OR 2.20, 95% CI: 1.77–2.74, *p =* 8.99 × 10^−13^). Regarding Q126X, the T allele was found on 4.7% of the chromosomes from the gout patients compared with 1.7% of the chromosomes from the controls (OR 2.91, 95% CI: 1.49–5.68, *p* = 1.57 × 10^−3^). The results of the association study, shown in Table 1, demonstrate that 141K and 126X were significantly associated with an increased risk of gout, whereas the frequency of the A allele of V12M appeared to be significantly decreased in gout patients (18.3%) compared with controls (29%) (OR 0.55, 95% CI: 0.43–0.71).

### 2.2. Haplotype Analysis

We performed a 3-SNP haplotype analysis (in the order V12M, Q126X and Q141K). The haplotype frequencies in the gout group were compared with the control population and all of the frequencies <0.03 were ignored in the analysis. We found that the frequencies of the GCA, GTC, GCC and ACC haplotypes were 0.481, 0.044, 0.292 and 0.165, respectively, among the gout patients and 0.289, 0.017, 0.404 and 0.271, respectively, among the controls. The GCA and GTC haplotypes were more frequently present in cases than in controls and could be regarded as risk haplotypes (ORs 2.3 and 2.71, respectively) (Table 2).

### 2.3. Association Analysis of ABCG2 Genotype Combinations in Gout Patients

Recently, it has been shown that genotyping for only the two dysfunctional variants, 126X and 141K, is sufficient to estimate the severity of ABCG2 dysfunction [16,18,19], which is strongly related to an increased risk of gout. To determine whether the association between ABCG2 dysfunction and gout could be replicated in a Chinese population, the patients were then divided into four groups using a genetically estimated ABCG2 function as follows: full function, 3/4 function (mild dysfunction), 1/2 function (moderate dysfunction) or ≤1/4 function (severe dysfunction). Table 5 shows the genotypes and estimated functions of ABCG2 in the 352 male gout cases and 350 controls.

The odds ratio was 2.40 (95% CI: 1.69–3.42; *p* = 1.00 × 10^−6^) in the 3/4 ABCG2 transport function group and 5.51 (95% CI: 3.46–8.77; *p* = 1.12 × 10^−13^) in the 1/2 function group. A modest increase in gout risk was observed in genotype combinations with ≤1/4 function (OR 5.90, 95% CI: 2.56–13.58; *p* = 8.47 × 10^−6^), and up to 6.2% of gout patients had this genotype. In contrast, only 2.3% of the normal males had the same genotype combinations (Table 3). Additionally, genotype combinations with full ABCG2 function were detected in 46.6% of the normal subjects but in only 21.6% of the gout patients with other risk factors for gout. These findings suggest that the combination of the 126X and 141K variants is a risk factor for developing gout.

## 3. Discussion

This study is the first to examine the possible role of *ABCG2* variants, which have previously been found to be associated with gout, in terms of their genetic susceptibility to gout in the Han Chinese population. We found that the Q141K, Q126X and V12M alleles were strongly associated with gout in Chinese males. The risk of gout was significantly increased by ABCG2 dysfunction, and even a mild dysfunction (3/4 function) conferred an increased risk of gout (OR 2.40).

Genome-wide association studies have identified several new and common genetic factors that affect serum uric acid levels. Most of these genetic factors are involved with the urate transporters located in the epithelial cells of the renal proximal tubules [10,20,21]. Among them, *SLC22A12*, *SLC2A9* and *ABCG2* are the most strongly associated with regulating the serum urate concentration [22]. Previously, we and others examined the SNPs *SLC22A12* and *SLC2A9*, and their associations with gout or hyperuricemia in different Chinese populations [23–26]. However, the relationship between common defects in ABCG2 function and the risk of gout has not yet been fully characterized in Han Chinese patients.

In addition to the absorptive transporter genes *SLC22A12* and *SLC2A9*, *ABCG2* is a secretory urate transporter gene. ABCG2 is expressed in the brush border membrane of the proximal tubules of the kidneys and plays a role in the apical secretion of urate [16]. Additionally, the transporter is also abundantly expressed in the apical membrane of epithelial cells in the small intestine and liver, suggesting a possible role in the extrarenal excretion of uric acid [27] and enhancing its regulatory role in the efflux of urate.

Consistent with the genetic susceptibility identified in gout patients in a cohort of Japanese individuals [18], we observed that the rare alleles of both the 141K and 126X SNPs of *ABCG2* were associated with an increased risk for gout, whereas the minor A allele in V12M had a protective effect on susceptibility to gout. The Q141K SNP has been extensively studied; it has been found to impair protein-nucleotide binding stability [28], which has been linked to hyperuricemia in a variety of populations [29]. This variant is found with low frequency in individuals of African-American (2%–5%), European (11%–14%), Hispanic (10%) or Middle Eastern (13%) descent, but it is found at high levels in individuals of Chinese (35%) or Japanese (35%) descent [30]. In this study, we found allele frequencies of 49.6% and 30.9%, respectively, in gout patients and normal controls, which are similar to a previous report on Northern Han Chinese [31]. These findings strongly support a similar genetic trait in the North Han population living in Shandong Province and the Han population in Shanghai.

The other nonfunctional variant, Q126X, is consistently observed in certain Japanese and Korean cohorts [18,32]. However, it is absent in Caucasian and African-American groups [33,34]. These findings reflect the diversity of the Q126X and Q141K distributions in different ethnic populations, which may explain the different prevalence of gout in Chinese and Caucasian populations.

Among the 352 patients with gout, Q126X heterozygous (*n =* 33) mutations were found that revealed that non-functional 126X dramatically increased gout risk (OR 2.91). The half-functional 141K also increased gout risk (OR 2.20). Matsuo *et al*. [16,18] reported that the genotype combination of Q126X and Q141K is a clinically important biomarker for predicting gout risk in the Japanese population. We analyzed the relationship between ABCG2 transport dysfunction and gout and found that dysfunctional ABCG2 is responsible for approximately 78.4% of gout cases. Moreover, the risk of gout is markedly increased by severe ABCG2 dysfunction, conferring an adjusted OR of 5.90. Thus, ABCG2 dysfunction is also a major cause of gout in Han Chinese individuals.

## 4. Materials and Methods

### 4.1. Patients

A total of 352 male gout patients and 350 gout-free normal male controls were recruited from Huashan Hospital of Fudan University. The diagnosis of gout was based on the 1977 American College of Rheumatology diagnostic criteria. Information regarding the medical history, condition and family history of the subjects was obtained from a medical interview of each subject at the time of enrollment. The serum BUN, creatinine and uric acid levels were measured using a Clinical Analyzer 7600 (Hitachi High-Technologies, Tokyo, Japan). The clinical features of the individuals enrolled in the study are summarized in Table 4. All of the subjects provided their written informed consent to participate. The study protocol was approved by the Ethics Committee of Huashan Hospital.

### 4.2. Genotyping ABCG2 with HRM

Genomic DNA was isolated from the peripheral blood lymphocytes of each patient using a QIAamp DNA Blood Kit (Qiagen, Valencia, CA, USA) according to the manufacturer’s instructions. Primer sequences were designed to amplify a small fragment surrounding the polymorphism and to avoid amplifying other sequence variations. All of the oligonucleotide primers were obtained from Sango Biotech Co. (Shanghai, China). We selected three functional *ABCG2* SNPs: V12M, Q126X and Q141K. *ABCG2* SNP genotyping was performed via HRM. The primer sequences are shown in Table 5. The reaction mixture consisted of 20 ng of genomic DNA, 1× polymerase chain reaction (PCR) buffer, 2.5 mM MgCl_2_, 200 nM each primer, 200 μM dNTPs, 5 μM SYTO 9, 0.5 U of HotStarTaq polymerase (Qiagen, Valencia, CA, USA) and PCR grade water in a 20 μL volume. PCR cycling and HRM were performed on a Rotor-Gene 6000™ (Corbett Research, Mortlake, New South Wales, Australia). All of the reactions were performed under the following conditions: one cycle of 95 °C for 15 min; 40 cycles of 95 °C for 30 s, 55 °C for 30 s and 72 °C for 30 s; a melt from 75 to 90 °C was also performed at intervals (ramps) of 0.1 °C/s. An HRM curve analysis was performed using the Rotor-Gene 6000 1.7 software (Corbett Research).

### 4.3. Sequencing

To confirm the genotyping results, PCR-amplified DNA samples were selected and examined via DNA sequencing. The sequencing primers were the same primers that were used in the HRM assay. The amplicons were gel purified using a QIAquick gel purification kit (Qiagen, Valencia, CA, USA). The DNA sequencing analysis was performed in an ABI PRISM 3130 genetic analyzer (Applied Biosystems, Foster City, CA, USA).

### 4.4. Statistical Analysis

The chi-squared test was used to analyze categorical data and compare the genotypes and allele frequency distributions. The odds ratios (ORs) and 95% confidence intervals (95% CIs) were calculated. Haplotype frequency analyses were performed using the SHEsis online software (http://analysis.bio-x.cn/myAnalysis.php). *p* Values less than 0.05 were considered statistically significant.

## 5. Conclusions

Because gout leads to a significantly impaired quality of life and imposes high life-long medical costs, early genetic testing of the *ABCG2* gene for protein dysfunction will help implement risk-management systems for gout. Developing an understanding of renal transporters will provide interesting targets for the development of future gout therapies. The function of some ABC transporter mutants has reportedly been rescued by pharmacological chaperones [35,36]. The recent findings on the roles of the *ABCG2* Q141K and Q126X polymorphism in gout may pave the way for pharmacological chaperones targeting ABCG2 as a potential new therapeutic target for gout. Moreover, risk assessment through genotyping only two SNPs with molecular technologies will provide a cost-effective screening strategy for personalized gout treatment that includes adequate prevention and effective therapy.

## Figures and Tables

**Table 1.** Association analysis of *ABCG2* variants in gout patients. MAF, minor allele frequency.

SNP	Genotype *									Allele Frequency Mode		
	
	Case				Control				*p-*Value	*p-*Value	OR	95% CI
	
	1/1	1/2	2/2	MAF	1/1	1/2	2/2	MAF				
Q141K	84	181	87	0.496	33	150	167	0.309	1.18 × 10^−11^	8.99 × 10^−13^	2.20	1.77–2.74
Q126X	0	33	319	0.047	0	12	338	0.017	1.31 × 10^−3^	1.57 × 10^−3^	2.91	1.49–5.68
V12M	16	97	239	0.183	35	133	182	0.290	3.67 × 10^−5^	2.55 × 10^−6^	0.55	0.43–0.71

* The minor allele was referred to as allele 1, and the major allele was referred to as allele 2. Allele 1 is A and allele 2 is C in Q141K. Allele 1 is T and allele 2 is C in Q126X. Allele 1 is A and allele 2 is G in V12M.

**Table 2.** Haplotype frequency analysis of V12M, Q126X and Q141K.

Allele			Frequency		*p-*Value	OR	95% CI
	
V12M	Q126X	Q141K	Gout	Control			
G	C	A	0.481	0.289	1.26 × 10^−13^	2.30	1.84–2.87
G	T	C	0.044	0.017	2.97 × 10^−3^	2.71	1.37–5.36
G	C	C	0.292	0.404	8.27 × 10^−6^	0.60	0.48–0.75
A	C	C	0.165	0.271	1.53 × 10^−6^	0.53	0.41–0.69

**Table 3.** Participants’ ABCG2 function levels.

Estimated Function	Genotype Combination		Number (%)		*p-*Value	OR	95% CI
	
	Q141K	Q126X	Gout	Control			
≤1/4 function	C/A	T/C	22 (6.2)	8 (2.3)	8.47 × 10^−6^	5.90	2.56–13.58
1/2 function	C/C	T/C	95 (27.0)	37 (10.5)	1.12 × 10^−13^	5.51	3.46–8.77
	A/A	C/C					
3/4 function	C/A	C/C	159 (45.2)	142 (40.6)	1.00 × 10^−6^	2.40	1.69–3.42
Full function	C/C	C/C	76 (21.6)	163 (46.6)		—	—

*p*-Value, OR and 95% CI for each ABCG2 dysfunction were obtained via comparisons with full function.

**Table 4.** Clinical and biochemical profile of gout patients and controls.

Index	Gout Patients	Controls	*p*-Value
Subjects (%)	352 (50.1%)	350 (49.9%)	
Age (year)	57.6 ± 14.0	56.6 ± 16.6	NS
BUN (mmol/L)	5.4 ± 1.9	5.5 ± 2.1	NS
Creatinine (μmol/L)	97.3 ± 15.7	96.1 ± 16.4	NS
Uric Acid (μmol/L)	456.4 ± 120.1	334.7 ± 88.7	<0.01

Data are expressed as the means ± standard deviation (S.D.).

**Table 5.** Primer sequences.

SNP ID	SNP Allele	Sequence (5′-3′)	Size
V12M	A/G	ATGGTATGGGCCATTCATTG ATGCCTTCAGGTCATTGGAA	250 bp
Q141K	A/C	ATGTTGTGATGGGCACTCTG CCACATTACCTTGGAGTCTG	158 bp
Q126X	C/T	GCTGCAAGGAAAGATCCAAG CAGCCAAAGCACTTACCCAT	163 bp
